# Hybrid Prepreg Tapes for Composite Manufacturing: A Case Study

**DOI:** 10.3390/ma15020619

**Published:** 2022-01-14

**Authors:** Mohanapriya Venkataraman, Jiří Militký, Alžbeta Samková, Daniel Karthik, Dana Křemenáková, Michal Petru

**Affiliations:** 1 Department of Material Engineering, Faculty of Textile Engineering, Technical University of Liberec, Studentska 2, 46117 Liberec, Czech Republic; jiri.militky@tul.cz (J.M.); alzbeta.samkova@tul.cz (A.S.); daniel.karthik@tul.cz (D.K.); dana.kremenakova@tul.cz (D.K.); 2 Department of Machinery Construction, Institute for Nanomaterials, Advanced Technologies, and Innovation, Technical University of Liberec, 46117 Liberec, Czech Republic; michal.petru@tul.cz

**Keywords:** glass multifilament, roving, prepreg tapes, hybrid, controlled spreading, tensile properties, Weibull distribution

## Abstract

The aim of this research was the preparation and characterization of hybrid prepreg tapes from glass multifilament roving (circular cross-section). The fiber, roving, and tape strength distribution was characterized by exploratory data analysis tools (especially quantile-quantile plot) and modeled by the three parameters’ Weibull distribution. For estimation of Weibull model parameters, the noniterative technique based on the so-called Weibull moments was used. It was shown that the prepared hybrid prepreg tapes prepared by controlled mechanical spreading technology developed by the authors improved mechanical tensile properties and can be used for the preparation of composites of complicated forms by robotic winding.

## 1. Introduction

**Figure 1:**
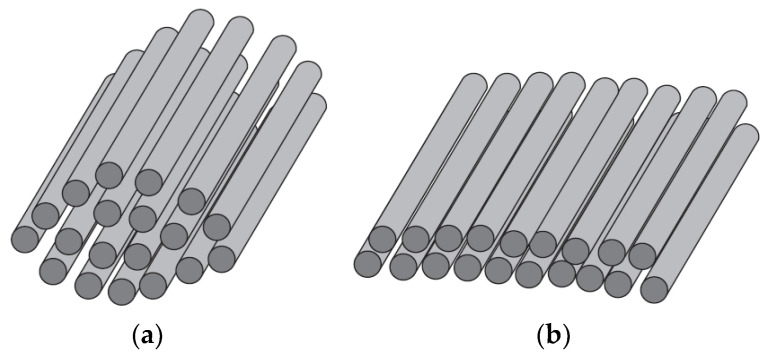
Geometry of (**a**) roving and (**b**) tape.

Globally, thermoset resins are the most commonly used composite materials with applications as matrix and glass fibers as cheap reinforcing agents [1,2,3,4,5,6,7,8,9]. The reinforcement bears the stresses [10,11] and the matrix ensures the cohesion of the composite and distributes and damps the impacts or stresses to protect the composite from the environment [12,13,14,15,16]. The design of composites depends on the choice of fibers’ orientation and stacking sequence as well [17]. Both the materials and manufacturing process influence the quality of the produced composites [18]. Advanced composites are commonly manufactured from prepreg materials where the reinforcing fibers are assembled in unidirectional tape form (Roving or tow) or as a woven fabric and then pre-impregnated with a resin [19,20,21,22,23]. Standard multifilaments can be simply replaced by spread tow tape (STT) with a rectangular cross-section (constant thickness), which evenly distributes the tensile stress. Their width is three times the diameter of the original circular multifilament (see Figure 1).

**Figure 2:**
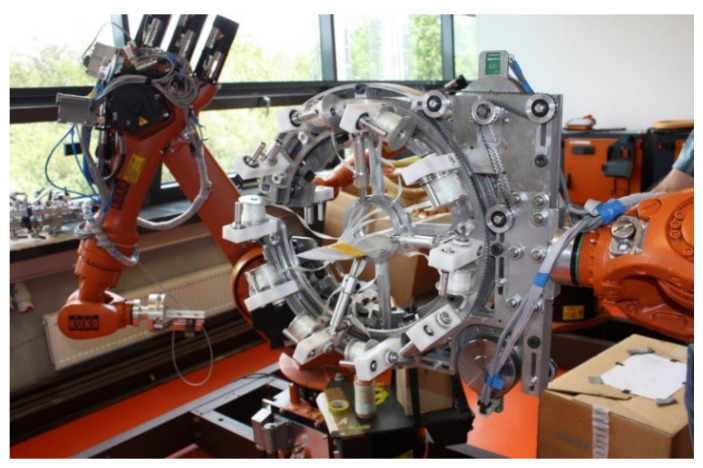
Robotized winding by using hybrid tapes (developed at TUL).

The individual filaments are much more closely arranged and straighter. They behave like a bunch of identical long parallel threads. This leads to better utilization of the strength of individual filaments and a reduction in the thickness (by 20–30%) and, hence, the density of the composites [20,21]. Prepregs tapes are being increasingly used in high-performance applications in the composites’ industry and also in other sectors. The application for the creation of composites with complicated shapes by robotized winding is relatively novel (see Figure 2).

The main aim of this study was to describe a simple technology of preparation of hybrid straight tape (rectangular cross-section) from a glass fiber multifilament (nearly circular cross-section) and the epoxy matrix by a prototype device for controlled mechanical spreading and straightening. We prepared the new hybrid tape suitable for robotic winding and described the properties of the material. We used industrially prepared roving available from different producers and showed how to change a cross-sectional shape and straightness to obtain better material. The device for the creation of the tape was proposed and checked in our lab. Therefore, the results presented are novel. The glass fiber multifilament roving from a Slovak supplier was used. The simple, idealized model of the spreading process based on the limited honeycomb arrangement of fibers in the roving cross-section is described. Tensile properties of individual fibers, roving, and tape were comprehensively analyzed, and the prediction of strength distribution was carried out by using advanced statistical tools.

## 2. Experimental

### 2.1. Materials Used

A glass multifilament roving StarRow 1200 from a Slovak supplier, Johns Manville Slovakia, a.s., Trnava, Slovakia was selected as reinforcement. The typical properties of these filaments provided by the supplier are given in Table 1.

The hybrid tape was prepared by the controlled spreading technology in the Technical University of Liberec. The hybrid tape specifications are given in Table 2.

The glass fibers are made of aluminosilicate E-glass in accordance with ASTM D 578. Mechanically spread SLT rovings were coated with a CHS-EPOXY 200 V 55 epoxy resin dispersion with a catalyst and pre-cured at 80 °C for 24 h.

### 2.2. Preparation of Tapes

**Figure 3:**
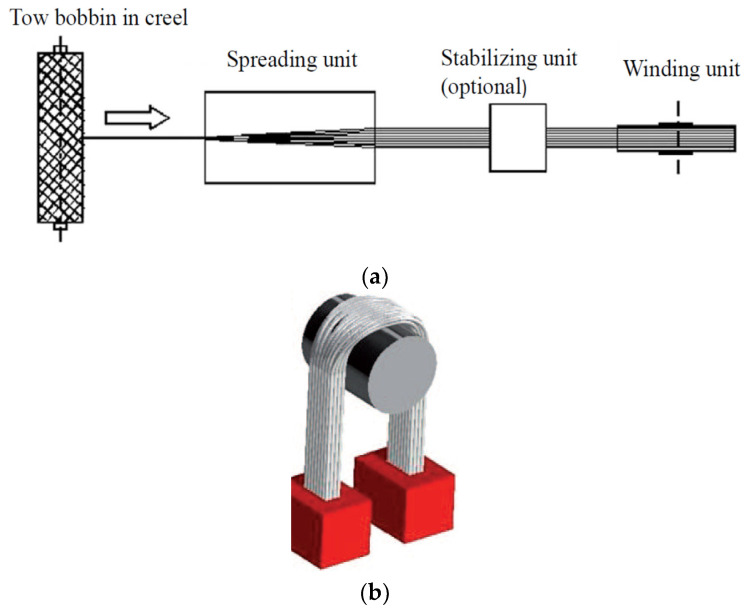
(**a**) Typical spreading system; (**b**) principle of mechanical spreading [18].

Spread-tow technology is based on the geometric rearrangement of fibrous tow into a thinner, flatter, and more oriented rectangular tape with increased load-carrying efficiencies (see Figure 3) [20].

**Figure 4:**
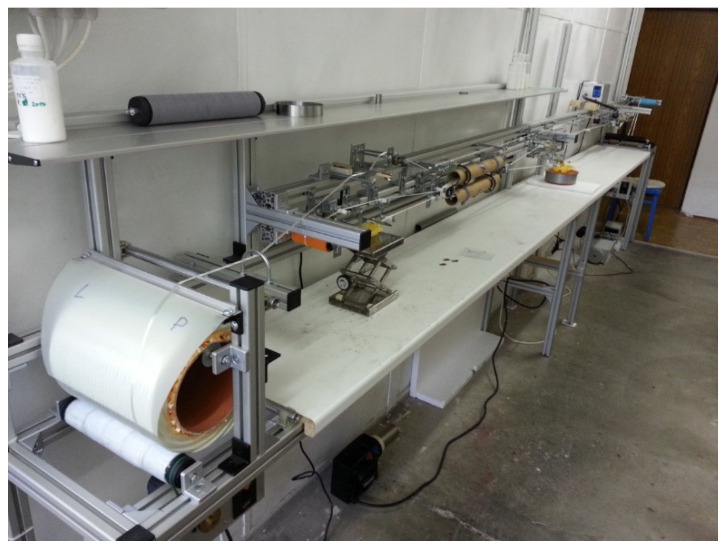
Semi-operating device for preparing a prepreg (© Global Science Press) [20].

Preparation of SLT was carried out in a pilot plant spreading unit built by Večerník s.r.o. company, Pěnčín, Czech Republic. Further development of this unit was carried out at the Faculty of Textile Engineering (see Figure 4). The principle is passive mechanical spreading around a rotating circular bar in a spreading unit (see Figure 3a,b). The unit also includes an element that will provide a constant strain of roving [24]. The overall path of roving before the wrap of the prepreg takes about 15 m.

In the first part, roving passes through the smooth steel rod system to ensure an even separation of the individual fibers.

The matrix phase in the hybrid SLT is made from a mixture of acrylate polymers and epoxy-silane, which are suitable for epoxy resin [13]. To ensure a sufficient epoxy matrix content, three epoxy dispersion-based coating stations were used in the line. At each of the stations, the impregnated roving was dried at elevated temperatures (80–120 °C). In terms of penetration into the fibrous structure, the first deposit is the most important. Further deposits are mainly due to the layers being interconnected with each other in the formation of the composite. After the last coating of the epoxy dispersion with the catalyst, cross-winding was performed on a paper roll.

### 2.3. Characterization and Test Methods

Scanning electron microscopy (SEM) was used to determine the microscopic characteristics of the roving and prepreg glass filament tapes. Images were performed on Sigma (Zeiss, Jena, Germany) equipment. The tensile properties were measured by a 1000 N (Instron instrument) head with pneumatic jaws and rubber, a clamping length of 200 mm, a jaw velocity of 50 mm/min, i.e., a strain rate of 0.25/min, a tape thickness of 0.16 mm (differences without epoxy), and a width of 4 mm. The tensile properties of fibers were measured in a Vibrodyn 400 instrument. For the prediction of strength distribution, the noniterative method of Weibull moments was selected.

## 3. Spreading Process

Wilson [25] studied the mechanical mode of spreading fibrous bundles, which could be extended to predict the mechanically induced spreading of E-glass fiber bundles [26].

**Figure 5:**
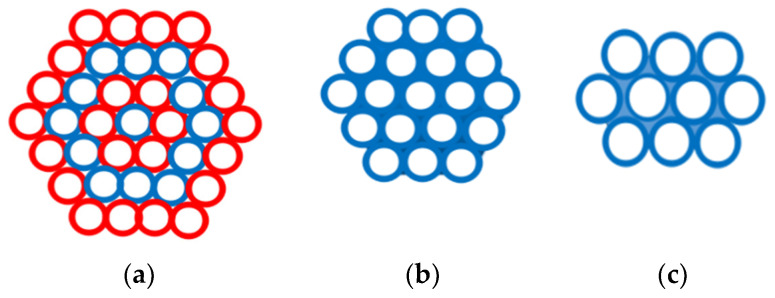
Honeycomb structure [27]. (**a**) 1 + 6+12 + 18 + …m (*i*). (**b**) Close honeycomb. (**c**) Open honeycomb. Limit packing density μ_hm_ =0.907.

The cross-section geometry of spread roving composed of circular fibers can be predicted from idealized structural arrangements. The simple, idealized spreading process assumes a limited honeycomb arrangement of fibers in the roving cross-section. In the so-called close honeycomb structures, the filaments are arranged in hexagonal concentric layers (see Figure 5).

In the first concentric layer of this structure there is only one filament, in the second layer there are six filaments, and in the *i*th layer, where *i* = 2, 3, etc., the number of filaments (m) can be determined from the Equation m (*i*) = 6 (*i* − 1). The structure containing *l* layers is composed of a total of *n* = 3*l*^2^ − 3*l* + 1 filaments. The number of layers *l_n_* in a close honeycomb structure is related to the total number of filaments *n* [27].

(1) $$l_{n}=0.5+\sqrt{n/3-1/12}$$

The circle diameter *D_n_* is the same area as the area circumscribed to the close honeycomb structure composed from filaments with diameter *d* [27].

(2) $$D_{n}=2d\left[\sqrt{n/3-1/12}-0.5+1/(2\cos30^{{}^{\circ}})\right]\sqrt{3\cos30^{{}^{\circ}}/\pi }$$

Roving with the fineness *T* [tex] composed from individual filaments with fineness *T* [tex] has a total number of filaments of *n_T_ = T/t*. If *n_T_ = n,* the tow can ideally follow a full-close honeycomb structure. The fineness of roving *T* is the product of the cross-section area of filaments S and glass density *ρ*. The diameter *D_T_* [mm] of ideal circular roving is then expressed in the form:

(3) $$D_{T}=\sqrt{\frac{4T}{\pi \mu \rho }}$$

**Figure 6:**
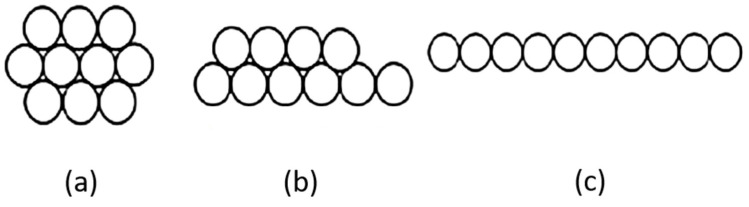
Idealized spreading process (**a**) a = 4d, b = 3d (**b**) a = 6d, b = 2d (**c**) a = 10d, b = d [27].

Here, the packing density for a compact honeycomb structure is *µ* = *µ_lim_* = 0.907 and for real filaments is *µ* about 0.7. For tow with fineness 1200 tex and glass density *ρ* = 2580, kg m^−3^ is then for a compact honeycomb *D_T_* = 0.808 mm and for real multifilaments *D_T_* = 0.9198 mm. During flattening the most optimal arrangement according to the compact honeycomb structure is maintained (see Figure 6).

The width of the multifilament *a* and thickness *b* are varied depending upon the number of layers. Relative width *α*, relative thickness *β*, and relative flatness *γ* (*γ* ≥ 1) are defined as $\alpha =a/D_{T};\;\beta {=b/D}_{T};\;\gamma =\alpha /\beta =a/b$.

There are two types of geometrical assumptions of spreading leading to the prediction of the relation between relative width and relative thickness.

A. Assumption of the constant area during spreading in combination with Kemp racetrack cross-section of tape leads to the relation [28]:

(4) $$\alpha =\left[\pi /4-\beta^{2}\left(\pi /4-1\right)\right]/\beta$$

B. Assumption of constant perimeter during spreading in combination with Kemp racetrack cross-section of tape leads to the relation [29]:

(5) α=[π−β(π−2)]/2

**Figure 7:**
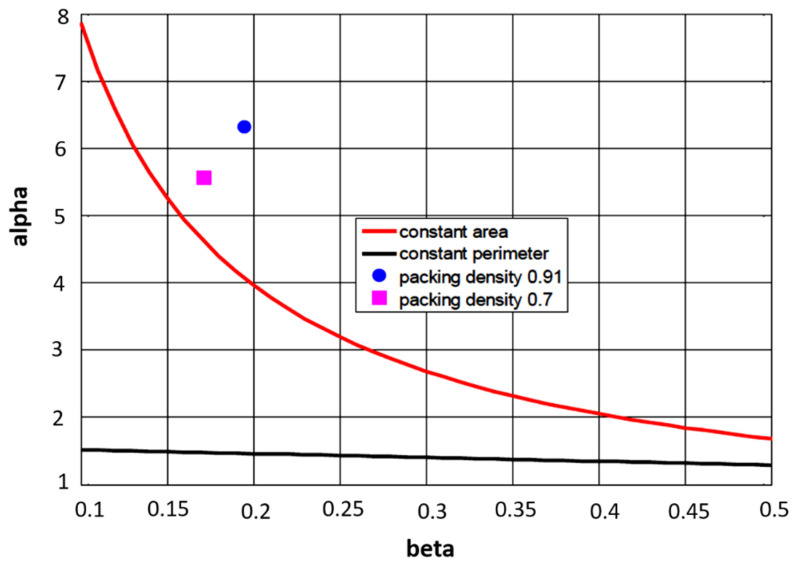
Dependence of tape width on thickness under different assumptions.

The dependence of *α* on *β* calculated from Equations (4) and (5) is shown as curves in Figure 7.

Let us have a tow (SL) of fineness 1200 tex composed from 2376 filaments with a close honeycomb structure. From Equation (1), the total number of layers is 29 including the central one. In the outer 29th layer, there are 107 filaments. The diameter of tow for a honeycomb is *D_T_* = 0.808 mm and for real multifilaments it is *D_T_* = 0.9198 mm. For filament fineness of 0.505 tex, the corresponding diameter is 0.0159 mm. The spread tow (SLT) has the same shape characteristics as tow SL. The corresponding relative width and thickness for both packing densities are given in Table 3.

Based on the assumption of the constant area during spreading, for *β* = 0.196 and 0.172, the calculated width is *α_c_* from Equation (4) for different packing densities through *D_T_*. It is visible that for *µ* = 0.7, the values of *α* and *α_c_* are closer (see also points in Figure 7).

## 4. Results and Discussion

### 4.1. Hybrid Tape Geometry

**Figure 8:**
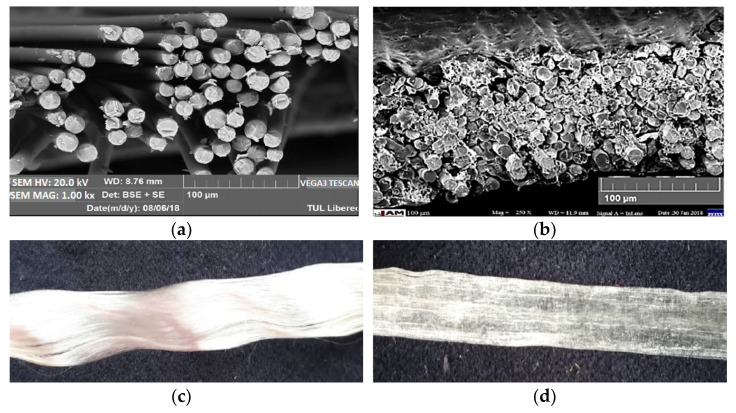
Material SL and SLT (**a**) glass filaments cross-section, (**b**) prepreg tape cross-section, (**c**) longitudinal view of roving, (**d**) longitudinal view of prepreg tape.

SEM was used to determine the microscopic characteristics of the glass filament prepreg tapes. It can be observed from Figure 8 that prepreg tapes are approximately rectangular in cross-section and are slightly undulating. The roving is slightly wavy; the straps are straight.

Individual filaments in SL materials were obtained by direct separation from roving.

### 4.2. Fiber Strength Distribution

**Figure 9:**
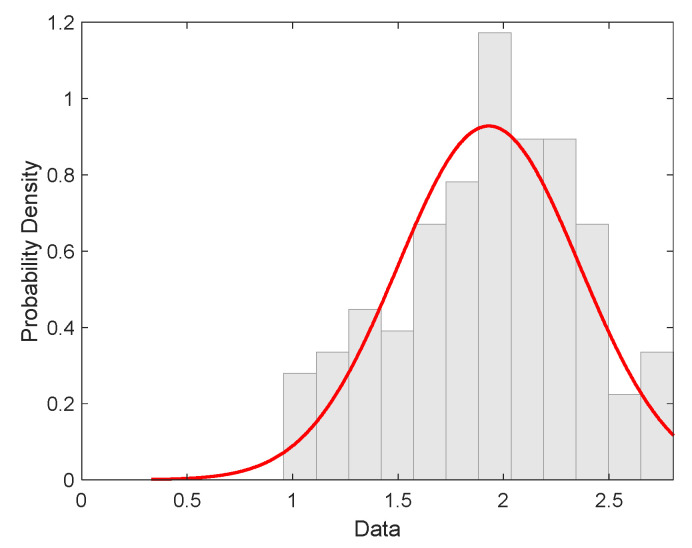
Histogram characterized by experimental distribution of filament strength (red curve is the probability density of normal distribution).

The main goal of modeling and statistical analysis was to specify the strength distribution and estimate the relevant parameters based on experimental strengths. The fibers separated manually from SL materials were tested on a tensile testing machine under standard conditions. Tensile loads were measured at a clamping length of 10 mm at an elongation rate of 10 mm/min. The load data were converted to break stress (strength, MPa). Then, 120 measurements were used for analysis and parameter estimation. The histogram of the experimental distribution of fiber strength is shown in Figure 9. The red curve is a pdf of the normal distribution with parameters’ sample mean and variance calculated from data by standard procedure (see Table 4) [29]. The histogram is showing a nearly symmetric distribution.

Basic statistical characteristics of the strength of individual fibers are given in Table 4.

**Figure 10:**
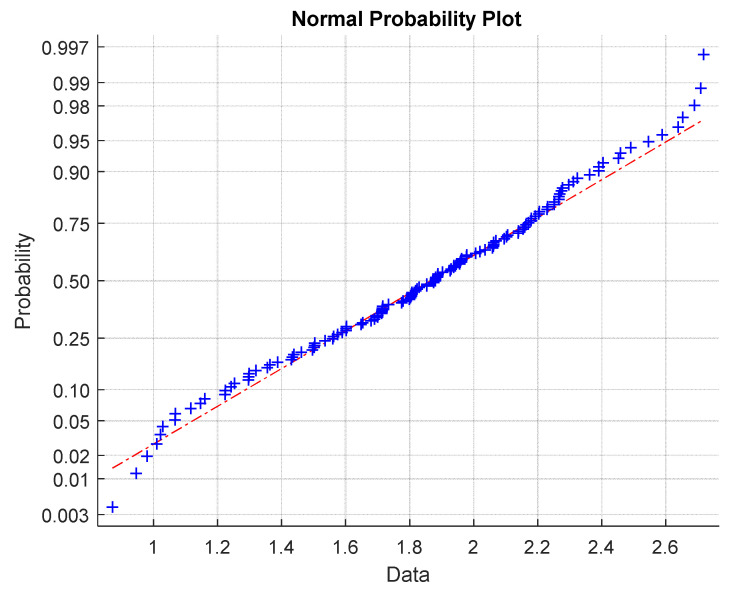
Q-Q graphs for data normality verification (red line is the ideal shape for normal distribution).

The quantile-quantile graph, which compares quantiles of experimental distribution (estimated simply by ordered experimental stress values arranged in ascending manner i.e., $\sigma_{B(1)}\leq \sigma_{B(1)}\leq ....\leq \sigma_{B(N)}$) with quantiles of selected theoretical distribution (here normal distribution), is used for checking the quality of the approximation of experimental distribution by the selected theoretical one. In the case when the experimental distribution is well approximated by the theoretical one, the Q-Q graph should be in the form of a straight line. For rigorous testing, there exist many characteristics. Therefore, a simpler correlation coefficient was used. The quantile-quantile (Q-Q) graph for assessing normality [29] (see Figure 10) shows slight systematic differences from the assumption of data normality near the lower and upper tails.

### 4.3. Roving Strength Distribution

Roving was tested on a tensile tester under standard conditions. Breaking loads were measured at a clamping length of 200 mm. The load data were converted to the breaking stress (strength). Then, 120 measurements were used for analysis and parameter estimation. The basic statistical characteristics of strength are given in Table 5.

Three parameters’ Weibull distribution was used as a model to analyze the strength distribution of roving and hybrid tape [23]. Parameters (*A*, *B*, *C*) of the Weibull distribution were estimated from experimental strengths by special moments. The three parameters are denoted as *C* > 0, the shape parameter responsible for the skew of the distribution; *B* > 0, the scale parameter; and, finally, *A* > 0, the shift parameter, which is also a lower bound). The distribution function of three parameters’ Weibull distribution is given by:

(6) $$F\left(\sigma \right)=1-\exp\left(-{\left(\frac{\sigma -A}{B}\right)}^{C}\right)$$

For the estimation of the parameters of Equation (6), plenty of different methods are used (see [29,30,31,32]). They are iterative and their solution for a non-zero threshold *A* is not in all cases successful. The non-iterative method based on the so-called Weibull moments *m_r_* defined by relations

(7) $$m_{r\;}=\overset{N-1}{\underset{i=0}{\sum }}{\left(1-\frac{i}{N}\right)}^{r}(\sigma_{f\left(i+1\right)-}\sigma_{f\left(i\right)})$$

is more reliable. In Equation (7), symbol $\sigma_{f(i)}$ means the *i*th order statistics, i.e., the *i*th smallest value of the breaking strength in the sample. Order statistics can be simply obtained by sorting experimental data in ascending order; for example, *i* = 0 is formally $\sigma_{f\left(0\right)}=0$. This technique can be used for the rough estimation of strength distribution parameters. Shape parameter *C* can be estimated from relation [24].

(8) $$C=\frac{\ln2}{\ln\left(m_{1}-m_{2}\right)-\ln\left(m_{2}-m_{4}\right)}$$

For the estimation of the threshold (lower limiting strength), *A* is valid as

(9) $$A=\frac{m_{1}\;m_{4}-m_{2}^{2}}{m_{1}+m_{4}-2m_{2}}$$

and we can estimate the scale parameter *B* in the form

(10) $$B=\frac{m_{1}-A}{\Gamma \left(1+1/C\right)}$$

where Γ(*x*) is Gamma function. These equations were here used for parameters *A*, *B*, and *C* estimation.

**Figure 11:**
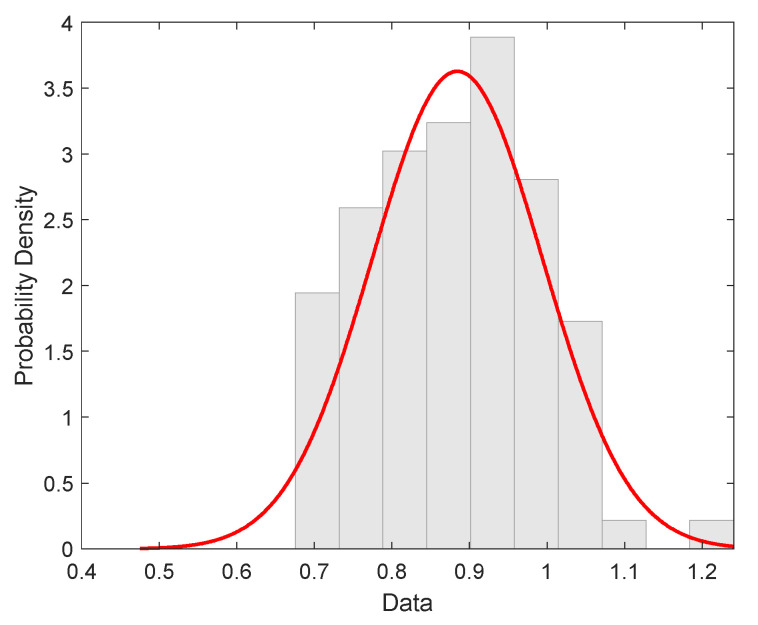
Experimental distribution of roving strength (red curve is the probability density of normal distribution).

The Histogram of the experimental tow strength distribution of filaments is shown in Figure 11.

**Figure 12:**
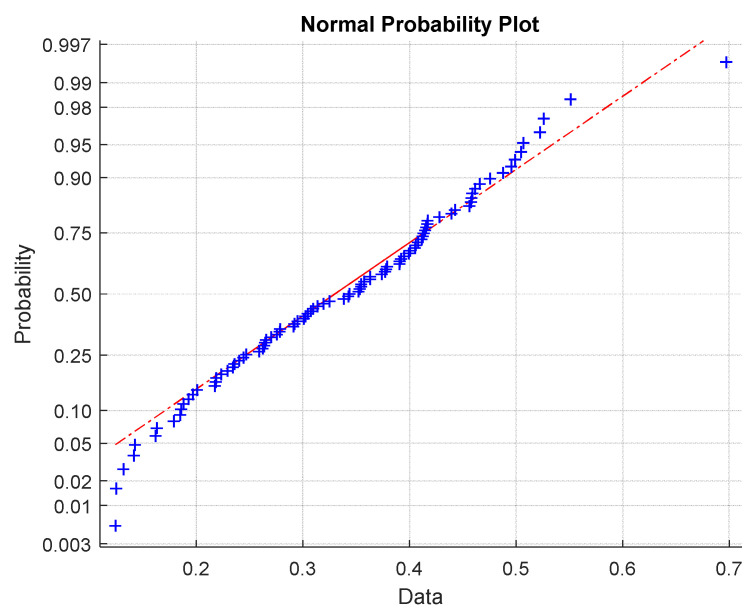
Q-Q graph for data normality verification (red line is an ideal shape for normal distribution).

Q-Q graphs to verify normal distribution (see Figure 12) showed slight differences in the end region from the assumption of data normality.

### 4.4. Hybrid Tapes Strength Distribution

The Hybrid Tapes were tested on a tensile tester under standard conditions. The breaking loads were measured at a clamping length of 200 mm. The load data were converted to the breaking stress (strength). A total of 25 measurements of SLT were made (simulating a real sample size in practice). The Basic statistical characteristics of the tape are given in Table 6.

**Figure 13:**
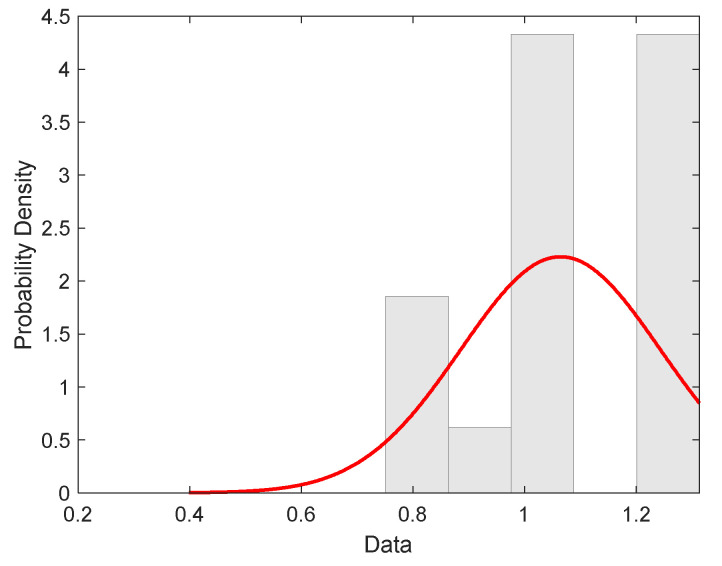
Experimental distribution of tape strength (red curve is the probability density of normal distribution).

The average strength of SLT must be higher than SL, indicating the validity of the mixing rules (the strength in the composite exceeds the strength of the strongest component). The experimental distribution of strength is shown in Figure 13.

**Figure 14:**
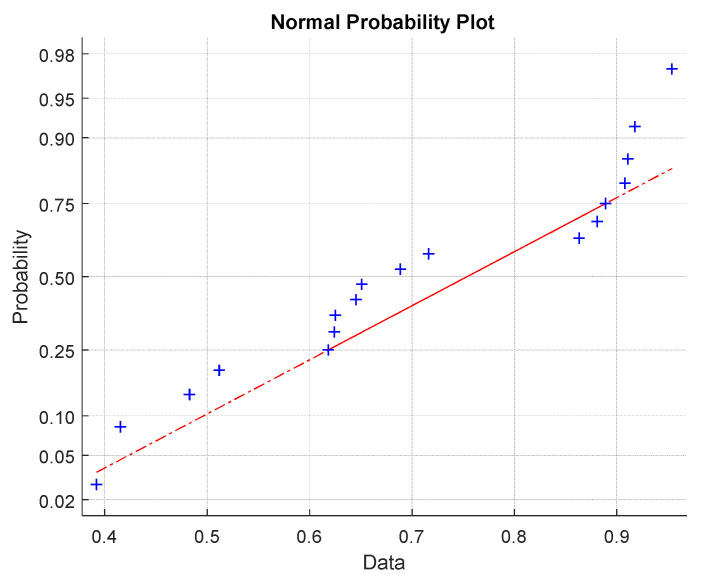
Q-Q graph for data normality verification (red line is the ideal shape for normal distribution).

Due to the small number of SLT samples, the constant non-parametric probability density function estimation based on the histogram was not very useful. The Q-Q graph for normal distribution (see Figure 14) showed differences in the tail regions from the assumption of normality. The assumption that the glass filaments break firstly (have lower breaking elongation than matrix phase) enabled us to predict tape strength from a simple mixture rule:

(11) $$\sigma_{T}=v_{f}\;\sigma_{B}+\left(1-v_{f}\right)E_{m}\;\varepsilon_{B}$$

where *E_m_* is the matrix modulus, *v_f_* is the volume fractions of the fiber, and *ε_B_* is the breaking strain of the fiber bundle, which is related to the bundle strength *σ_B_* *= E_f_* *ε_B_*, where the tensile modulus of the bundle is identical to that of the fiber.

The tape strength *σ_T_* is the sum of contribution of the fibrous phase *σ_Tf_* and the matrix phase *σ_TM,_* given as:

(12) $$\sigma_{T}=\sigma_{Tf}+\sigma_{TM}=v_{f}\;\sigma_{B}+\left(1-v_{f}\right)\frac{E_{m}}{E_{f}}\;\sigma_{B}$$

The contributions of the fiber (*v_f_* = 0.642) and matrix phases to the hybrid tape strength calculated from the simple rule of mixture are given in Table 7.

Due to the approximative normality of roving strength *σ_B_*, the tape strength is *σ_T,_* as well as being approximately normally distributed, and for mean tape strength ${\bar{\sigma }}_{T}$ it is valid [16].

(13) $${\bar{\sigma }}_{T}=\left(v_{f}+\frac{\left(1-v_{f}\right)E_{m}}{E_{f}}\right){\bar{\sigma }}_{B}$$

Corresponding standard deviation *S_T_* has the form:

(14) $$S_{T}=\left(v_{f}+\frac{\left(1-v_{f}\right)E_{m}}{E_{f}}\right)S_{B}$$

where ${\bar{\sigma }}_{B}$ and *S_B_* are the mean fiber bundle strength and its standard deviation. These relations are showing the prediction of tape strength basic statistical characteristics from characteristics of roving and matrix. Table 8 shows the calculated mean tape strength and corresponding standard deviations from parameters of roving and filament strengths (calculated from Equations (15) and (16)).

In comparison with experimental results (see Table 6), the agreement of mean values was visibly moderate, but the predicated standard deviations were highly underestimated. The reason is probably a higher error component due to experimental errors (uncertainty). The mean and variance of Weibull distribution are related to parameters *A*, *B*, and *C* by relations

(15) $$E\left(\sigma_{f}\right)=B\Gamma \left(1+\frac{1}{C}\right)+A\;\;$$

(16) $$D\left(\sigma_{f}\right)=B^{2}\left[\Gamma \;\left(1+\frac{2}{C}\right)-\Gamma^{2}\left(1+\frac{1}{C}\right)\right]$$

In the case of no validity of mixture rule, it is possible to adopt the hypothesis that filaments can be broken in matrix phase repeatedly into smaller parts until their lengths will be over the critical length *l_c_* [30]

(17) $$l_{c}={\left(\frac{r_{f}\;\left(\frac{4}{3}B\right)\Gamma \left(1+\frac{1}{C}\right)}{\tau_{y}}\right)}^{C/\left(1+C\right)}$$

where *r_f_* is the fiber radius and $\tau_{y}$ is the yielding shear strength of the matrix adjacent to the interface or that of the fiber–matrix interface, whichever is less. This modification is crucial so that the effect of fiber–matrix interaction can be included. The mean ${\bar{\sigma }}_{T}$ and standard deviation *S_T_* of tape strength can be, therefore, simply calculated by replacing bundle strength and standard deviation by ${\bar{\sigma }}_{B}\;\left(l_{c}\right)$ and $S_{B}\;\left(l_{c}\right),$ i.e., by modification of parameter *B* due to different lengths (replacing *l_f_* by the critical length *l_c_*) [31,32].

## 5. Conclusions

The proposed device for spreading can be used for the preparation of hybrid tapes with enhanced mechanical properties. It is apparent from the SEM images that the glass fibers in the active hybrid SLT tapes were evenly spaced along with the tape and evenly surrounded by the epoxy resin to form the required rectangular cross-section. The quality of the interface looked good. The fiber strength distribution can be successfully predicted by the Weibull distribution. The tensile strength distribution of rovings can also be described using the Weibull distribution. For predictive purposes, the strength characteristics (mean and standard deviation) can be calculated from the parameters (*A*, *B*, *C*) of the Weibull distribution. It was found that the SLT hybrid tape developed has improved mechanical tensile properties (see Table 7) useful for example robotic winding.

## Figures and Tables

**Table 1:** Typical Properties of Johns Maneville Co. Glass Rendering SL (Slovakia).

Properties	Unit	Value
Fineness	Tex	1200
Filament diameter	µm	16
Tensile strength	N	450

**Table 2:** Specifications of selected materials containing glass.

Material	Standard	Resin	Manufacturer	Acronym
Roving	ASTM D 1505	-	Slovakia	SL
Hybrid tape	ASTM C 338	CHS-EPOXY 200 V 55	Czech Republic–TUL and Večerník	SLT

**Table 3:** Idealized geometry of SLT.

Material	Width ‘a’ [mm]	Thickness ‘b’ [mm]	*D_T_* [mm]	*α*	*β*	*α_c_*
SLT µ = 0.9	5.122	0.158	0.81	6.34	0.196	4.05
SLT µ = 0.7	5.122	0.158	0.92	5.57	0.172	4.603

**Table 4:** Basic statistical properties of fiber strength.

Material	Mean Value [GPa]	Standard Deviation [GPa]	Coefficient of Variation [%]
Fibers from SL	1.93	0.43	22.37

**Table 5:** Roving strength characteristics and parameters of the Weibull distribution.

Material	Mean Value [GPa]	Standard Deviation [GPa]	Threshold A [GPa]	Weibull Form C [-]	Weibull Scale B [GPa]
SL	0.88	0.11	0.57	3.10	0.35

**Table 6:** Tape strength characteristics and Weibull distribution parameters.

Material	Mean Value [GPa]	Standard Deviation [GPa]	Threshold A [GPa]	Weibull Form C [-]	Weibull Scale B [GPa]
SLT	1.06	0.18	0.36	4.49	0.77

**Table 7:** Components of hybrid tape strength.

Material	Tape ${\bar{\sigma }}_{T}$ [GPa]	Fiber ${\bar{\sigma }}_{T}$ [GPa]	Matrix ${\bar{\sigma }}_{T}$ [GPa]	$E_{m}/E_{f}$
SLT	1.064	0.568	0.496	0.178

**Table 8:** Predicted basic statistical characteristics of tape.

Material	Mean Pred. [GPa]	Standard Deviation Pred. [GPa]
SLT	1.1835	0.0117
